# Long-Term Reproductive Outcomes After Palmer-Type Neosalpingostomy in Hydrosalpinx: A Seven-Year Real-World Cohort Study

**DOI:** 10.3390/jcm14228043

**Published:** 2025-11-13

**Authors:** Laurențiu Augustus Barbu, Nicolae-Dragoș Mărgăritescu, Liliana Cercelaru, Tiberiu Stefăniță Țenea Cojan, Mădălina Costinela Stănică, Irina Enăchescu, Ana-Maria Țenea Cojan, Valentina Căluianu, Gabriel Florin Răzvan Mogoș, Liviu Vasile

**Affiliations:** 1 Department of Surgery, Railway Clinical Hospital Craiova, University of Medicine and Pharmacy of Craiova, 200349 Craiova, Romania; laurentiu.barbu@umfcv.ro (L.A.B.); tiberiu.tenea@umfcv.ro (T.S.Ț.C.); valentina.andronache@yahoo.com (V.C.); gabriel.mogos@umfcv.ro (G.F.R.M.); 2 Department of Surgery, Emergency County Hospital, University of Medicine and Pharmacy of Craiova, 200349 Craiova, Romania; vliviu777@yahoo.com; 3 Department of Embryology and Anatomy, University of Medicine and Pharmacy of Craiova, 200349 Craiova, Romania; liliana.cercelaru@umfcv.ro; 4 Independența VitaPlus Hospital, 200738 Craiova, Romania; stanica.madalina@yahoo.com (M.C.S.); irinaenachescu@gmail.com (I.E.); 5 Faculty of Medicine, University of Medicine and Pharmacy of Craiova, 200349 Craiova, Romania; anamariatenea0324@gmail.com

**Keywords:** hydrosalpinx, neosalpingostomy, tubal infertility, pelvic adhesions, reproductive outcomes

## Abstract

**Background:** Hydrosalpinx is a major cause of female infertility, but the long-term outcomes of Palmer-type neosalpingostomy remain insufficiently documented. **Methods:** We retrospectively analyzed 160 women with primary or secondary infertility and laparoscopically confirmed hydrosalpinx who underwent Palmer-type neosalpingostomy at a single specialized center in Romania (2018–2024). Patients were enrolled consecutively, and disease severity was classified intraoperatively using standardized criteria. The primary outcome was clinical pregnancy; secondary outcomes included live birth, miscarriage, ectopic pregnancy, recurrence, and time to conception. **Results:** The clinical pregnancy rate was **33.8%**, intrauterine/live birth rate **25.6%**, miscarriage rate **3.8%**, and ectopic pregnancy rate **4.4%**. Recurrence occurred in **21.2%** of cases. Outcomes were strongly influenced by hydrosalpinx severity and pelvic adhesions: women with mild disease achieved the highest pregnancy rates, whereas those with severe adhesions had poor prognosis. Neither age, AMH, nor laterality were independent predictors. Median time to pregnancy was **9 months**. **Conclusions:** Palmer-type neosalpingostomy can achieve satisfactory reproductive outcomes in selected women, particularly those with mild hydrosalpinx and no adhesions. This study provides long-term real-world evidence from Eastern Europe, complementing international literature and emphasizing the importance of individualized patient selection.

## 1. Introduction

Infertility affects 10–15% of couples worldwide, with female factors accounting for approximately one-third of cases [1]. Tubal pathology contributes to 20–30% of female infertility, and hydrosalpinx represents one of its most severe forms [2,3]. Defined as a fluid-filled dilatation of the fallopian tube secondary to distal occlusion, hydrosalpinx most commonly arises from pelvic inflammatory disease, endometriosis, prior pelvic surgery, or ectopic pregnancy [4].

Hydrosalpinx exerts a detrimental impact on fertility in both natural conception and assisted reproduction, with untreated cases showing significantly reduced implantation and pregnancy rates in in vitro fertilization (IVF) cycles [4,5]. The underlying mechanisms include mechanical embryo washout, embryotoxicity, impaired endometrial receptivity, and an increased risk of ectopic pregnancy [2,5].

Surgical management is the standard of care before IVF. Salpingectomy remains the gold standard, supported by randomized controlled trials and systematic reviews demonstrating improved pregnancy outcomes [2]. Recent meta-analyses confirm that salpingectomy and proximal tubal occlusion yield comparable live birth outcomes, although salpingectomy may provide higher fertilization rates and ongoing pregnancy rates [6,7]. Neosalpingostomy, originally described by Palmer, preserves tubal anatomy and allows the possibility of natural conception [2], but is limited by high recurrence rates and an elevated risk of ectopic pregnancy [4].

Despite these drawbacks, neosalpingostomy continues to play a role in selected patients, particularly younger women with bilateral hydrosalpinx who wish to conceive naturally. Best candidates are women with mild-to-moderate tubal damage, preserved ovarian reserve, and no extensive pelvic adhesions, while those with severe disease are more suitable for IVF. Yan et al. reported spontaneous live birth in nearly 28% of women after neosalpingostomy, compared with none after salpingectomy [2]. Conversely, other studies documented poor outcomes and high recurrence, underscoring the need for further evaluation [4].

First described by Palmer in 1947, the Palmer-type neosalpingostomy was conceived as a microsurgical procedure aimed at restoring fimbrial function and maintaining natural fertility potential. Unlike traditional salpingostomy, Palmer’s technique emphasizes atraumatic handling, precise eversion of the fimbrial edges, and meticulous preservation of the tubal mucosa, thereby improving postoperative patency and reducing adhesion formation. This microsurgical approach aligns with modern principles of fertility-sparing surgery and remains particularly valuable in settings where access to assisted reproductive technologies is limited.

Previous studies have reported pregnancy rates ranging from 20% to 35% following neosalpingostomy, depending on the severity of tubal disease and postoperative adhesions [4,8]. More recent analyses have compared neosalpingostomy with salpingectomy in women with hydrosalpinx, showing comparable cumulative live birth rates in selected cases, though long-term data remain limited [2,6,7]. These findings suggest that Palmer-type neosalpingostomy continues to have a role in individualized reproductive surgery, particularly for younger patients with mild-to-moderate tubal damage and preserved ovarian reserve.

**Figure 1:**
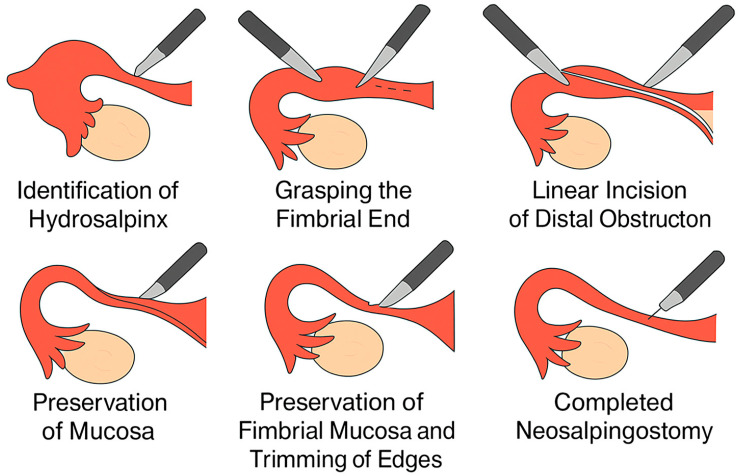
Simplified schematic of Palmer-type neosalpingostomy (original illustration created by the authors).

Palmer-type neosalpingostomy preserves tubal anatomy and allows the possibility of natural conception. A schematic representation of the Palmer-type neosalpingostomy is shown in Figure 1.

Given the conflicting evidence, long-term data on Palmer-type neosalpingostomy are needed. This study evaluated seven-year reproductive outcomes in women with primary and secondary infertility due to bilateral hydrosalpinx, adding to the debate on the role of this fertility-preserving procedure.

## 2. Materials and Methods

### 2.1. Study Population

This retrospective study included 160 women with primary or secondary infertility and bilateral hydrosalpinx who underwent Palmer-type neosalpingostomy at Independența Vita Plus Hospital, Craiova, Romania, between January 2018 and December 2024. The main indication for surgery was infertility evaluation and management, with all patients undergoing laparoscopy as part of the diagnostic and therapeutic workup for tubal factor infertility. In most cases, hydrosalpinx was suspected based on ultrasound or hysterosalpingography and subsequently confirmed intraoperatively. The study followed an all-comers design, meaning that every eligible patient treated during the study period was included, without selection based on disease severity, comorbidities, or reproductive plans. Enrollment was strictly based on fulfilling the predefined eligibility criteria, independent of surgeon preference or other factors. Although the initial inclusion criteria targeted patients with bilateral hydrosalpinx, a small proportion of unilateral cases were identified intraoperatively and retained in the final analysis to reflect real-world clinical practice and allow comparison by laterality. The inclusion criteria were aged between 18 and 45 years, laparoscopically confirmed bilateral hydrosalpinx, and infertility lasting at least 12 months. The exclusion criteria were unilateral hydrosalpinx, because natural conception remains possible through the contralateral tube and would represent a major confounding factor; history of previous tubal surgery, which could alter pelvic anatomy and independently affect fertility outcomes; pelvic malignancy, which could confound reproductive prognosis; and incomplete clinical records or patients lost to follow-up. All male partners underwent semen analysis as part of the infertility workup prior to surgery. Only couples with semen parameters within the standard clinical reference range were included. Cases with abnormal or subnormal semen profiles were excluded to avoid confounding effects on reproductive outcomes.

The severity of hydrosalpinx was classified intraoperatively using a validated scoring system widely applied in reproductive surgery (Table 1).

This system evaluates the degree of tubal dilatation, fimbrial agglutination, and peritubal adhesions, allowing categorization into mild, moderate, or severe disease. For the purposes of the present analysis, patients were stratified into three groups (mild, moderate, severe) based on the intraoperative findings. All patients were systematically followed at 1, 3, 6, and 12 months after surgery and annually thereafter, until conception or until the end of the observation period. Recurrence of hydrosalpinx was defined as the reappearance of an anechoic tubular fluid-filled structure in the adnexal region consistent with a dilated fallopian tube, confirmed on transvaginal ultrasound during follow-up. When ultrasound findings were equivocal, pelvic MRI or second-look laparoscopy was performed for confirmation. A minimum diameter of ≥10 mm on ultrasound was used as the threshold to define recurrence. All ultrasound assessments were performed by experienced gynecologic sonographers blinded to the surgical details. The overall follow-up rate was 100%, as patients with incomplete records or lost to follow-up were excluded before final analysis. To ensure a homogeneous study population with comparable reproductive prognosis, only women with bilateral disease and at least 12 months of infertility were included. The chosen age range of 18 to 45 years corresponds to the internationally accepted reproductive window where ovarian reserve and fertility potential are clinically relevant. Recruiting all patients from a single specialized center ensured uniformity in surgical technique and postoperative management, although this may limit the external generalizability of the results. Prior to surgery, all patients received individualized counseling regarding the available management options for hydrosalpinx, including neosalpingostomy versus salpingectomy followed by in vitro fertilization (IVF). The final decision was made after discussing the advantages, risks, and accessibility of each approach, taking into account patient age, disease severity, and personal preference. In our setting, access to IVF is limited and self-funded, and many patients preferred a fertility-preserving surgical option allowing the possibility of natural conception.

### 2.2. Surgical Procedure and Postoperative Management

Palmer-type neosalpingostomy was performed laparoscopically using microsurgical techniques with atraumatic tissue handling and meticulous hemostasis to minimize adhesion formation. All neosalpingostomies were performed jointly by two senior reproductive surgeons who operated together in all cases, ensuring consistency and minimizing variability in surgical technique. In women with polycystic ovary syndrome (PCOS), ovarian drilling was performed during the same surgical session when clinically indicated.

A schematic representation of the Palmer-type neosalpingostomy is shown in Figure 1.

All patients received postoperative oral contraceptive treatment, as recommended by their attending gynecologist, to promote complete tubal mucosal healing and to minimize the risk of early postoperative conception before restoration of normal fimbrial function. In most cases, the treatment was prescribed for 2–3 months postoperatively, in accordance with standard microsurgical recommendations for tubal repair and established clinical practice. This short course was not associated with any apparent delay in conception, as the median time to pregnancy was 9 months. The exact regimen and duration were individualized, but all followed current guideline-based recommendations. Preoperative microbiological evaluation was performed in all patients using endocervical and vaginal swabs collected before surgery. Samples were processed in the hospital’s microbiology laboratory, and culture results were used to guide targeted antibiotic therapy when indicated.

### 2.3. Outcomes

The primary outcome was the clinical pregnancy rate (CPR), defined as the presence of a gestational sac with or without fetal cardiac activity, confirmed on transvaginal ultrasound. Secondary outcomes included intrauterine pregnancy rate (IUPR), live birth rate, ectopic pregnancy, miscarriage, recurrence of hydrosalpinx, and time to conception.

### 2.4. Statistical Analysis

Data were initially processed using Microsoft Excel (Microsoft Corp., Redmond, WA, USA) with XLSTAT version 2024.3 (Addinsoft SARL, Paris, France). Statistical analyses were subsequently performed using SPSS version 25.0 (IBM Corp., Armonk, NY, USA). Continuous variables were expressed as mean ± standard deviation (SD) or median (interquartile range, IQR), while categorical variables were summarized as absolute numbers and percentages. Group comparisons (primary vs. secondary infertility; unilateral vs. bilateral hydrosalpinx; severity subgroups) were conducted using Chi-square test or Fisher’s exact test for categorical variables, and Student’s *t*-test or Mann–Whitney U test for continuous variables, as appropriate.

Univariate and multivariate logistic regression analyses were applied to identify independent predictors of intrauterine/live birth pregnancy outcomes, and results were reported as odds ratios (OR) with 95% confidence intervals (CI). Multivariate models included potential confounders such as age, AMH, presence of pelvic adhesions, comorbidities, and concomitant ovarian drilling in PCOS patients. Comorbidities, including PID history and infection status (positive microbial cultures), were also considered as covariates.

Kaplan–Meier survival analysis was used to estimate time to clinical pregnancy, with non-pregnant cases censored at the last follow-up. A two-sided *p*-value < 0.05 was considered statistically significant. To account for multiple testing across predefined subgroup comparisons, the Holm–Bonferroni correction was applied; sensitivity analyses using the Benjamini–Hochberg false discovery rate (FDR) yielded consistent results.

A priori power analysis was performed to verify the adequacy of the sample size. For a two-sided α = 0.05, detecting a difference between a baseline clinical pregnancy rate of 20% (typical for poor prognosis hydrosalpinx) and the observed 33.8% rate yielded statistical power > 0.99. Similarly, the effect size for disease severity (Cohen’s w ≈ 0.59) provided >0.99 power for the χ^2^ test across severity subgroups. These calculations confirm that the sample size of 160 patients was sufficient for the study’s primary and secondary objectives.

No imputation methods were applied for missing data; only complete datasets were included in the final analysis.

### 2.5. Ethical Approval

The study was approved by the Institutional Ethics Committee of Independența Vita Plus Hospital, Craiova, Romania (approval no. 1/2025). All procedures were performed in accordance with the ethical standards of the institutional research committee and with the Declaration of Helsinki. Patient data were anonymized, and all participants provided written informed consent for their medical records to be used in research, in accordance with institutional and ethical standards.

## 3. Results

### 3.1. Baseline Characteristics

A total of 160 patients were included in the analysis, of which 52.5% presented with primary infertility and 47.5% with secondary infertility. Although bilateral hydrosalpinx was an inclusion criterion, some patients were later classified as unilateral due to intraoperative findings or postoperative imaging revisions. The mean age was 35.2 years (±4.5), with a mean AMH level of 2.15 ng/mL (range 0.3–4.5). Bilateral hydrosalpinx was observed in 53.1% of cases, while pelvic adhesions were present in 59.4%. Ovarian drilling was performed in 42.5% of patients with PCOS. Additional pelvic pathologies included chronic pelvic inflammatory disease (chronic PID), polycystic ovary syndrome, uterine fibroids, and metabolic disorders (Table 2).

**Figure 2:**
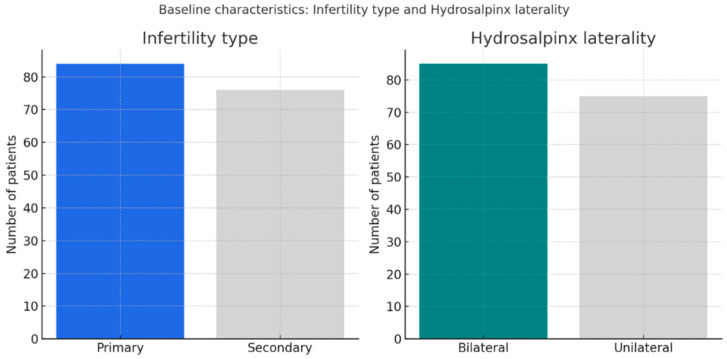
Distribution of infertility type and hydrosalpinx laterality. Legend: Primary and secondary infertility were equally distributed (52.5% vs. 47.5%), while bilateral hydrosalpinx was slightly more frequent (53.1% vs. 46.9%).

Figure 2 shows the distribution of infertility type and hydrosalpinx laterality in the study cohort. Primary and secondary infertility were nearly equally represented (52.5% vs. 47.5%), while bilateral hydrosalpinx was slightly more common than unilateral disease (53.1% vs. 46.9%). This distribution reflects a well-balanced study population, with a modest predominance of bilateral involvement.

### 3.2. Associated Pathologies and Procedures

Associated pelvic pathologies such as endometriosis (8 cases, 3.7%), uterine fibroids (36 cases, 16.7%), and polycystic ovary syndrome (66 cases, 30.6%) were recorded in our cohort, together with obesity (31 cases, 14.4%), hypothyroidism (27 cases, 12.5%), type 2 diabetes mellitus (5 cases, 2.3%), and other conditions. Although these comorbidities are well-recognized contributors to infertility, statistical analysis in our study did not show a significant association with intrauterine/live birth pregnancy outcomes.

Overall, 48 patients (30%) had positive microbial cultures, most frequently *Mycoplasma hominis* (21.3%), *Gardnerella vaginalis* (20%), and *Atopobium/Prevotella* (18.1%). Less common isolates included *Ureaplasma* spp. (15%) and *Chlamydia trachomatis* (5%).

Ovarian drilling was performed in 42.5% of patients with PCOS. This procedure did not show a significant correlation with intrauterine/live birth pregnancy outcomes. This variability reflects real-world practice, as ovarian drilling was performed only when clinically indicated (e.g., anovulatory PCOS with poor response to medical treatment).

### 3.3. Pregnancy Outcomes

Following Palmer-type neosalpingostomy, the clinical pregnancy rate was 33.8%. After excluding ectopic pregnancies (4.4%) and miscarriages (3.8%), the intrauterine/live birth pregnancy rate was 25.6%. These outcomes align with previously reported series, which describe clinical pregnancy rates of 20–35% after neosalpingostomy (Table 3).

**Figure 3:**
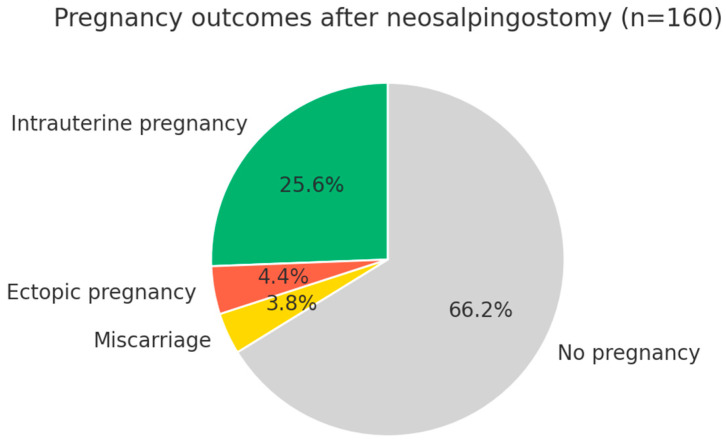
**Pregnancy outcomes after Palmer-type neosalpingostomy (n = 160)**. Legend: The clinical pregnancy rate was 33.8% (54 patients). After excluding ectopic pregnancies (4.4%) and miscarriages (3.8%), the intrauterine and intrauterine/live birth pregnancy rate was 25.6% (41 patients). Two-thirds of the patients (66.2%) did not achieve pregnancy during follow-up.

Figure 3 illustrates pregnancy outcomes after Palmer-type neosalpingostomy. Intrauterine pregnancies occurred in 25.6% of cases, while 4.4% resulted in ectopic pregnancy and 3.8% in miscarriage. The majority of patients (66.2%) did not conceive during follow-up, underscoring both the reproductive potential and the inherent limitations of the procedure, including persistent risks of ectopic pregnancy and pregnancy loss.

Pregnancy rates did not differ significantly between primary and secondary infertility, nor between unilateral and bilateral hydrosalpinx. The clinical pregnancy rate was 33.3% in women with primary infertility and 34.2% in those with secondary infertility, while intrauterine/live birth pregnancy rates were 25.0% and 26.3%, respectively. Similarly, clinical pregnancy occurred in 36.5% of women with bilateral hydrosalpinx and 30.7% with unilateral disease, with comparable intrauterine/live birth pregnancy rates (24.7% vs. 26.7%). Statistical analysis confirmed no significant differences across groups (all *p* > 0.05), and the odds ratio for clinical pregnancy according to laterality (0.77) indicated that tubal side involvement was not an independent predictor of outcome (Table 4).

### 3.4. Complications and Recurrence

Recurrence of hydrosalpinx was identified in 21.2% of patients. Among patients with recurrence, none achieved spontaneous pregnancy. Most were subsequently counseled to undergo IVF as the recommended treatment, while a minority declined further reproductive interventions. No repeat neosalpingostomy was performed during the follow-up period. Ectopic pregnancies occurred in 4.4% of cases, and miscarriages were recorded in 3.8% (Table 5).

#### Recurrence by Severity

Overall, hydrosalpinx recurred in 34/160 patients (21.2%). Recurrence rates by intraoperative severity were 18.5% (5/27) in moderate, 20.0% (9/45) in mild, and 22.7% (20/88) in severe disease, indicating a numerically higher propensity to recur in severe cases.

### 3.5. Predictive Factors 

Univariate logistic regression showed no significant association between intrauterine/live birth pregnancy and age, AMH, laterality, recurrence, or medical history (Table 6). In contrast, pelvic adhesions were strongly associated with reduced pregnancy likelihood (OR = 0.28, 95% CI: 0.13–0.59, *p* = 0.001). Ovarian drilling was performed in 42.5% of patients with PCOS. In multivariate analysis, drilling did not show a significant independent association with intrauterine or live birth pregnancy outcomes. Neither PID history nor infection status emerged as independent predictors of intrauterine/live birth pregnancy once adhesions were accounted for. Pregnancy outcomes were also analyzed according to the performance of concomitant ovarian drilling in women with polycystic ovary syndrome. Clinical pregnancy occurred in 32.4% of women who underwent drilling and in 34.8% of those who did not (*p* > 0.05). Similarly, live birth rates were 29.4% and 31.5%, respectively (*p* > 0.05). These differences were not statistically significant, suggesting that ovarian drilling did not independently influence reproductive outcomes after neosalpingostomy.

To further evaluate the impact of age, patients were stratified into two subgroups (<35 years, n = 47; ≥35 years, n = 113). Clinical pregnancy rates were 31.9% and 34.5%, respectively, with no statistically significant difference between age groups (*p* > 0.05).

In the multivariate model adjusting for age and AMH, pelvic adhesions remained the only independent negative predictor of intrauterine/live birth pregnancy (OR = 0.28, 95% CI: 0.13–0.59, *p* = 0.001). Neither age (OR = 1.01, *p* = 0.797) nor AMH (OR = 0.98, *p* = 0.925) showed a significant association with outcomes, confirming the consistent detrimental impact of adhesions irrespective of patient age or ovarian reserve (Table 7). In our cohort, adhesions were most frequently associated with chronic pelvic inflammatory disease, but were also observed in women with endometriosis and in those with a history of prior pelvic surgery, all of which are recognized risk factors for adhesion formation.

**Figure 4:**
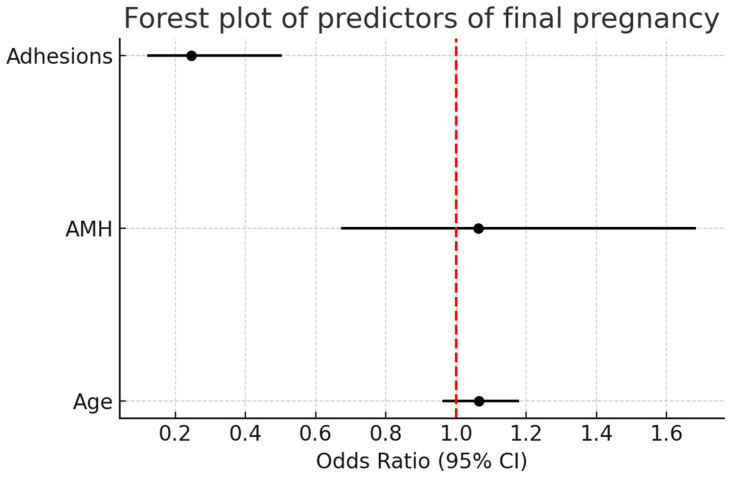
Forest plot analysis of predictors of intrauterine/live birth pregnancy. Pelvic adhesions were a significant negative predictor (OR = 0.25, 95% CI: 0.12–0.50, *p* = 0.001), while age and AMH showed no significant association. The red dashed vertical line represents the null value (OR = 1.0), indicating no effect.

Forest plot analysis (Figure 4) demonstrated that pelvic adhesions were a significant negative predictor of intrauterine/live birth pregnancy (OR = 0.25, 95% CI: 0.12–0.50, *p* = 0.001). By contrast, age and AMH showed no significant association, with odds ratios approximating 1 and confidence intervals crossing unity.

The sample size provided high statistical power for detecting large effect sizes (e.g., disease severity: w ≈ 0.59; χ^2^ = 56.1; df = 2; power > 0.99). Similarly, the difference between the observed CPR (33.8%) and a minimal clinically relevant rate of 20% would have been detected with power > 0.99 at α = 0.05. Therefore, the absence of significant associations for certain covariates (e.g., age, AMH) is unlikely to be solely explained by insufficient power.

### 3.6. Impact of Hydrosalpinx Severity

Intrauterine/live birth pregnancy rates (Table 8) were significantly associated with hydrosalpinx severity, with the highest rate observed in minor cases (73.3%), followed by moderate (25.9%) and severe (10.2%) hydrosalpinx (χ^2^ = 56.1, *p* < 0.001).

### 3.7. Time to Conception 

Kaplan–Meier analysis demonstrated that the median time to clinical pregnancy was **9.0 months** (IQR 8.0–12.0). As shown in Table 9, the cumulative incidence of pregnancy increased gradually over the first year after surgery, reaching **0.6%** at 3 months, **4.4%** at 6 months, **16.2%** at 9 months, **21.9%** at 12 months, and **25.6%** at 15 months. Non-pregnant cases were censored at the end of follow-up.

**Figure 5:**
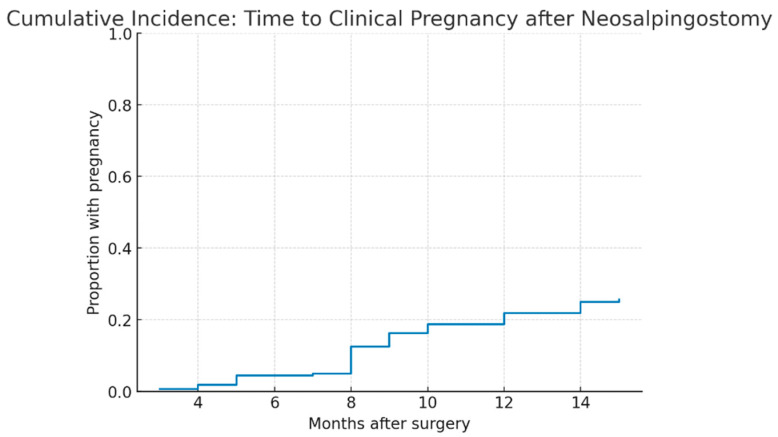
**Cumulative incidence of clinical pregnancy after Palmer-type neosalpingostomy**. Legend: Kaplan–Meier cumulative incidence curve of clinical pregnancy in the study cohort (n = 160). The cumulative proportion of patients achieving pregnancy increased gradually during follow-up, with a median time to pregnancy of 9 months (IQR 8–12). The curve plateaued after 12 months, and non-pregnant cases were censored at the end of follow-up.

The cumulative incidence of clinical pregnancy after neosalpingostomy is shown in Figure 5. The probability of pregnancy increased progressively during the first year and plateaued after 12 months.

## 4. Discussion

### 4.1. Study Overview

This retrospective study evaluated the efficacy of Palmer-type neosalpingostomy in women with primary and secondary infertility associated with bilateral hydrosalpinx over a seven-year period. Our study provides long-term data on neosalpingostomy; however, the absence of a comparator group limits direct comparison with outcomes after salpingectomy or IVF. The clinical pregnancy rate was 33.8%, with a final intrauterine/live birth rate of 25.6%, while ectopic pregnancy and miscarriage occurred in 4.4% and 3.8% of cases, respectively. These outcomes are consistent with previous series reporting pregnancy rates of 20–35% after neosalpingostomy [4,8]. For instance, Kasia et al. reported an intrauterine pregnancy rate of 26.1% with a 2.3% ectopic rate, whereas Bayrak et al. [4,8] described much lower success in women with severe tubal pathology. Collectively, these findings support neosalpingostomy as a fertility-preserving option in selected patients, with results largely determined by tubal morphology and the presence of adhesions.

Several gynecological and systemic comorbidities were present in our cohort, most frequently polycystic ovary syndrome (30.6%), pelvic inflammatory disease (27.8%), uterine fibroids (16.7%), obesity (14.4%), hypothyroidism (12.5%), and endometriosis (3.7%). Although none were independent predictors of outcome in our regression analysis, these conditions are well-recognized contributors to infertility and may have acted as confounding factors. Prior studies have demonstrated that comorbidities such as endometriosis and fibroids can impair implantation and increase miscarriage risk even after technically successful tubal repair [9,10,11,12]. Likewise, metabolic and endocrine disorders including obesity, diabetes, and thyroid dysfunction may compromise ovulation, endometrial receptivity, and embryo implantation [8,12,13,14,15].

Nearly half of the women with PCOS underwent concomitant ovarian drilling, a procedure known to improve spontaneous conception, particularly in younger women with favorable reproductive profiles [16,17,18]. Thus, some pregnancies in this subgroup may reflect a combined effect of drilling and tubal repair rather than the independent efficacy of neosalpingostomy. Furthermore, metabolic disorders frequently associated with PCOS (e.g., obesity, insulin resistance, diabetes) are recognized to negatively impact fertility [19,20,21,22]. These findings highlight the multifactorial determinants of reproductive success and the need for comprehensive management strategies, including lifestyle interventions and metabolic optimization alongside surgical treatment [20,23,24].

Taken together, these results underscore that the benefit of neosalpingostomy must be interpreted within the broader context of associated pelvic and systemic conditions. Larger prospective studies are required to clarify the independent contribution of these factors and refine patient selection for optimal outcomes.

### 4.2. Comparison with Previous Literature

Early studies reported poor outcomes after neosalpingostomy, with Bayrak et al. describing recurrence rates of up to 70% and only 5% intrauterine pregnancies in poor-prognosis women [4]. More recent series, however, suggest more favorable results. Yan et al. found that while salpingectomy achieved higher cumulative live birth rates and lower ectopic risk, almost 28% of women conceived spontaneously after neosalpingostomy [2,5]. Similarly, Kasia et al. reported a 28.5% overall pregnancy rate (26.1% intrauterine, 2.3% ectopic) in a large cohort of 402 women [8], and Milingos et al. observed cumulative intrauterine pregnancy rates of 29% [25]. Soumaila et al. documented a 32.5% pregnancy rate, with nearly 80% intrauterine conceptions, though fertility declined beyond the fourth postoperative year [14]. By contrast, Taylor et al. reported lower efficacy (24.5% intrauterine pregnancy, 16.5% ectopic) [26], and recurrence rates as high as 70% remain a concern in poor-prognosis groups [27].

Collectively, these findings highlight the heterogeneity of reported outcomes and underscore the importance of careful patient selection to optimize the benefits of neosalpingostomy.

Recent meta-analyses have reported live birth rates of approximately 32–38% per IVF cycle following salpingectomy for hydrosalpinx, compared with markedly lower rates (15–20%) in untreated cases [5,6,7,14]. Yu et al. similarly reported that tubal restorative surgery for hydrosalpinx can achieve spontaneous pregnancy and live birth rates comparable to those of IVF after salpingectomy in selected patients, particularly when tubal damage is mild and fimbrial mucosa is preserved [28]. In our cohort, the spontaneous live birth rate of 25.6% after neosalpingostomy approaches the lower bound of IVF outcomes reported after salpingectomy, although direct comparison is limited by differences in patient selection and study design. These findings suggest that in selected women with mild tubal disease, neosalpingostomy may provide a reasonable opportunity for natural conception, while IVF after salpingectomy remains the treatment of choice for severe hydrosalpinx.

### 4.3. Prognostic Factors

Disease severity emerged as the most decisive prognostic factor. Hao et al. reported intrauterine pregnancy rates of 50.5% in mild hydrosalpinx, 32.9% in moderate, and only 10.7% in severe cases [29]. Similarly, Kasia et al. found that rates reached 73.9% in women without adhesions but dropped to 8.8% in those with severe adhesions [8]. In our cohort, preserved ovarian reserve (mean AMH 2.15 ng/mL) and relatively younger age likely contributed to the favorable outcomes observed, consistent with prior studies showing better results in women under 35 years with adequate ovarian reserve [8,11]. By contrast, pelvic adhesions—present in nearly 60% of patients—were strongly associated with reduced pregnancy rates, aligning with evidence that extensive adhesions markedly compromise outcomes [8,30]. Age did not significantly influence reproductive outcomes in our cohort, consistent with previous studies indicating that tubal morphology and adhesions are more relevant prognostic factors than chronological age. This likely reflects the fact that the detrimental impact of PID and genital tract infections is largely mediated through adhesion formation, which was strongly associated with reduced pregnancy rates in our cohort.

The ectopic pregnancy rate was 4.4% (13% of all conceptions), reflecting a known limitation of tubal surgery: while neosalpingostomy restores patency, it cannot fully normalize tubal transport. This is comparable to reported ectopic rates of 10–15% [8,9]. An additional consideration is the management of women with PCOS, nearly half of whom underwent concomitant ovarian drilling during the same surgical session. Ovarian drilling is known to enhance ovulation and spontaneous conception [9,10,31]; however, only 42.5% of PCOS patients in our cohort received this intervention. This heterogeneity reflects real-world clinical practice but may have introduced a confounding effect, since some pregnancies in this subgroup could reflect a combined benefit of drilling and tubal repair rather than neosalpingostomy alone. Although nearly half of the women with PCOS underwent ovarian drilling, this factor was included in the multivariate model and did not emerge as an independent predictor of outcomes. Although nearly half of the women with PCOS underwent concomitant ovarian drilling, subgroup analysis showed no significant difference in clinical pregnancy (32.4% vs. 34.8%) or live birth rates (29.4% vs. 31.5%) compared with those without drilling (*p* > 0.05). Therefore, ovarian drilling did not appear to independently influence reproductive outcomes after neosalpingostomy, although a minor confounding effect cannot be fully excluded [8,32].

### 4.4. Time to Clinical Pregnancy

An important finding was the relatively short median time to clinical pregnancy (9 months), with most conceptions achieved within the first postoperative year and a plateau thereafter. This pattern is consistent with previous reports indicating that pregnancies after neosalpingostomy occur predominantly in the early postoperative period, with declining likelihood thereafter. Clinically, these results underscore the need for close follow-up during the first year and support timely referral to ART for women who remain infertile after 12–15 months, in order to avoid unnecessary delays in parenthood.

### 4.5. Postoperative Management

All patients received postoperative oral contraceptives to promote tubal healing and prevent early conception. Women with polycystic ovary syndrome (PCOS) often underwent concomitant ovarian drilling, which may have contributed to the observed pregnancy rates and represents a potential confounder. Patients with pelvic inflammatory disease (PID) were treated with targeted antibiotics based on culture and antibiogram to eradicate infection, limit further pelvic damage, and improve reproductive outcomes. Patients with endometriosis underwent conservative surgical excision or ablation of visible lesions during the neosalpingostomy procedure, followed by the same 2–3 month course of postoperative oral contraceptives as the rest of the cohort before attempting conception. Patients with pelvic inflammatory disease (PID) were treated with targeted antibiotics based on culture and antibiogram, and in our cohort PID was identified in 60 women (27.8%). These adjuvant measures reflect standard clinical practice; however, their individual impact on fertility outcomes could not be assessed separately due to the retrospective design of the study.

### 4.6. Role of Infections and Pelvic Adhesions

Approximately one-third of our patients had positive microbial cultures, most frequently *Mycoplasma hominis*, *Gardnerella vaginalis*, *Atopobium/Prevotella*, *Ureaplasma* spp., and *Chlamydia trachomatis*. These pathogens are established causes of pelvic inflammatory disease (PID), which is strongly associated with tubal obstruction and infertility [2,4,32,33,34]. *C. trachomatis* accounts for up to one-third of tubal infertility cases [2], while *Mycoplasma* and *Ureaplasma* contribute to chronic inflammation and adhesion formation [3,34]. Anaerobes linked to bacterial vaginosis (e.g., *Gardnerella*, *Prevotella/Atopobium*) may also predisposed to recurrent PID and hydrosalpinx [3,35,36]. In line with these mechanisms, Kasia et al. reported intrauterine pregnancy rates of only 8.8% in women with extensive PID-related adhesions [8].

In our cohort, a history of PID and the presence of these microorganisms were indirectly reflected by the high prevalence of pelvic adhesions (≈60%), which emerged as an independent negative predictor of pregnancy. This supports the pathophysiological model whereby chronic infection induces fibrosis, impairs tubal motility, and disrupts the peritoneal environment required for fertilization and implantation. Adhesion formation in these patients is largely explained by chronic inflammatory and fibrotic processes. Pelvic inflammatory disease induces persistent peritoneal inflammation and fibrosis, endometriosis promotes cyclical bleeding and scarring, and prior pelvic surgery may disrupt peritoneal surfaces, all of which facilitate adhesion development and impair tubal motility.

Overall, these findings emphasize that infectious and inflammatory factors decisively influence reproductive outcomes after Palmer-type neosalpingostomy. While the procedure restores tubal patency, its effectiveness is significantly reduced in the presence of extensive adhesions, underscoring the importance of PID prevention and early treatment to preserve fertility and maximize surgical benefit.

### 4.7. Primary Versus Secondary Infertility

Our cohort included nearly equal proportions of primary (52.5%) and secondary (47.5%) infertility, which strengthens the external validity of the findings. Secondary infertility is frequently related to prior pelvic infection or surgery [2,37], whereas primary infertility is more often associated with congenital anomalies or long-standing tubal disease [3]. Several studies, including Kasia et al., have reported higher pregnancy rates in women with secondary infertility following neosalpingostomy [8]. Although no statistically significant difference was observed between primary and secondary infertility, a slight numerical trend suggested potentially better outcomes among women with secondary infertility, consistent with previous reports.

### 4.8. Clinical Relevance

Although IVF with prior salpingectomy is regarded as the gold standard for hydrosalpinx management [1], Palmer-type neosalpingostomy preserves the possibility of natural conception, which remains important for women unwilling or unable to pursue ART [38]. In our cohort, a live birth rate of 25.6% highlights the clinical value of this approach in carefully selected patients. However, cumulative live birth rate (CLBR), which is considered a more clinically meaningful outcome in infertility research, could not be determined from our retrospective data, and this remains an important limitation when interpreting the long-term reproductive potential of neosalpingostomy. Because live birth data were recorded per pregnancy rather than per patient, cumulative live birth rate could not be accurately calculated. Based on available data, the observed live birth proportion (25.6%) likely approximates the lower boundary of the CLBR. Future prospective studies should systematically collect data on subsequent pregnancies and assisted reproduction outcomes to provide a more accurate estimation of cumulative fertility potential after neosalpingostomy. The 4.4% ectopic pregnancy rate, though lower than earlier reports [25], emphasizes the need for thorough counseling and close postoperative surveillance. Recurrence, observed in 21.2% of cases, also has important clinical and economic implications, as many of these women required counseling for IVF, and repeated interventions may offset the initial advantages of tubal-preserving surgery compared with upfront salpingectomy and IVF. This limitation underscores the need for prospective studies assessing not only recurrence but also subsequent reproductive pathways and cost-effectiveness. The clinical and economic impact of recurrence is not unique to reproductive surgery; similar challenges have been described in vascular surgery, where recurrent or progressive pathology significantly alters prognosis and long-term cost-effectiveness [38]. In resource-limited settings, neosalpingostomy may remain the only accessible fertility-preserving option [26,39,40,41,42,43]. Beyond clinical outcomes, economic and contextual factors are equally important when counseling patients. In high-income healthcare systems, the cost-effectiveness of neosalpingostomy may be questioned given the superior success rates of IVF after salpingectomy. However, in low- and middle-income settings where assisted reproduction remains inaccessible or unaffordable, tubal-preserving surgery offers a pragmatic and cost-efficient alternative that enables natural conception without the recurring expenses of ART. Patient selection should therefore consider not only disease severity and tubal morphology but also socioeconomic context, healthcare coverage, and personal preferences. Women with mild-to-moderate hydrosalpinx, preserved ovarian reserve, and no extensive adhesions are most likely to benefit, whereas those with severe disease or advanced age may be better served by IVF following salpingectomy.

Recurrence occurred across all severity strata but was most frequent in severe hydrosalpinx (22.7%), supporting the pragmatic view that Palmer-type neosalpingostomy has limited durability in advanced tubal damage. Clinically, women with mild–moderate disease may still benefit from tubal-preserving surgery, whereas those with severe lesions should be counseled for early IVF or salpingectomy prior to IVF.

### 4.9. Surgical Technique and Operator Experience

Surgical technique and operator experience are key determinants of tubal patency and long-term reproductive outcomes after Palmer-type neosalpingostomy [40]. Factors such as microsurgical precision, atraumatic tissue handling, and meticulous hemostasis are critical for success. In this retrospective study, however, these variables could not be objectively evaluated, and heterogeneity in surgical skill or intraoperative decision-making may have contributed to outcome variability. This limitation is inherent to retrospective designs and highlights the need for prospective studies with standardized surgical protocols to reduce confounding.

### 4.10. Strengths and Limitations

The main strengths of this study are the relatively large sample size, the extended seven-year follow-up, and the homogeneous definition of the study population, as only women with bilateral hydrosalpinx were included. This approach minimized heterogeneity and enabled a more reliable assessment of long-term reproductive outcomes compared with smaller or less focused series [8,40]. Another strength is that all procedures were performed in a single specialized center by two senior surgeons who operated jointly, ensuring a standardized surgical technique and consistent postoperative management. Male factor infertility was excluded, as all male partners presented normal semen parameters before surgery, thereby minimizing potential confounding effects related to male reproductive factors. The systematic recording of comorbidities and prognostic factors further allowed adjustment for potential confounders and facilitates meaningful comparison with other studies.

Several limitations should also be acknowledged. First, the retrospective design inherently restricts causal inference, and findings should be interpreted as associations rather than proof of causality. Second, the absence of a comparator group (e.g., salpingectomy or IVF) precludes any direct comparison of relative efficacy; accordingly, our results should be interpreted as descriptive rather than comparative. Third, cumulative live birth rate (CLBR), a clinically relevant endpoint in infertility research, could not be calculated due to the retrospective design and data structure; only live births per pregnancy were available. Fourth, severity classification relied on intraoperative assessment without formal interobserver validation, although standardized criteria and performance by a small surgical team minimized this risk. Fifth, a recurrence rate of 21.2% was observed, but its long-term implications on fertility and the eventual need for assisted reproduction could not be fully assessed. Time-to-recurrence was not captured in our retrospective dataset; therefore, only recurrence rates, not recurrence times, could be analyzed by severity. Finally, the generalizability of our findings may be limited, as only surgically managed women were included.

### 4.11. Future Directions

Future randomized controlled trials directly comparing Palmer-type neosalpingostomy with salpingectomy are essential to establish their relative efficacy and safety. In addition, predictive models that integrate tubal morphology, adhesion severity, and ovarian reserve markers could enhance patient selection and guide individualized treatment strategies. Long-term prospective studies should also evaluate recurrence, cumulative live birth rates, and the durability of restored fertility, thereby defining more precisely the role of neosalpingostomy in contemporary reproductive surgery. Further long-term follow-up is warranted to determine the cumulative live birth rate and to document reproductive outcomes after recurrence, including the use of IVF or repeat surgery.

### 4.12. Final Remarks

This study provides real-world, observational data from a large and homogeneous cohort of women with hydrosalpinx undergoing Palmer-type neosalpingostomy in Romania. Its main value lies in the descriptive characterization of long-term reproductive outcomes in a well-defined population, thereby complementing international literature. While salpingectomy prior to IVF remains the gold standard, these findings illustrate the potential role of neosalpingostomy as a fertility-preserving option in selected women, particularly in settings where access to assisted reproduction is limited.

## 5. Conclusions

Palmer-type neosalpingostomy can achieve meaningful pregnancy and live birth rates in women with hydrosalpinx, but outcomes are strongly influenced by disease severity and the presence of pelvic adhesions. The best candidates are women with mild tubal damage and no adhesions, whereas those with extensive adhesions or prior pelvic inflammatory disease are more likely to benefit from IVF. Neither age, ovarian reserve, nor laterality were independent predictors, while comorbidities and concomitant procedures may have acted as confounders. Careful patient selection, prevention and early treatment of genital infections, and adherence to standardized microsurgical protocols are essential to maximize safety and effectiveness.

## Figures and Tables

**Table 1:** Intraoperative classification of hydrosalpinx severity.

Severity	Tubal Dilatation	Fimbrial Status	Peritubal Adhesions
**Mild**	<2 cm	Minimal fimbrial agglutination	Absent or filmy, easily lysed
**Moderate**	2–3 cm	Partial fimbrial agglutination	Moderate, limiting mobility
**Severe**	>3 cm	Complete fimbrial agglutination	Dense, extensive, distorting tubo-ovarian anatomy

**Table 2:** Baseline characteristics of the study population (n = 160).

Characteristic	Value
**Total patients**	160
**Infertility type**	Primary: 84 (52.5%)/Secondary: 76 (47.5%)
**Age (years)**	Mean 35.2 ± 4.5 (SD)
**AMH (ng/mL)**	Mean 2.15 (range 0.3–4.5)
**Hydrosalpinx laterality**	Bilateral: 85 (53.1%)/Unilateral: 75 (46.9%)
**Pelvic adhesions**	Present: 95 (59.4%)/Absent: 65 (40.6%)
**Ovarian drilling (PCOS)**	Performed: 68 (42.5%)/Not performed: 92 (57.5%)
**Associated comorbidities**	PCOS: 66 (30.6%); PID: 60 (27.8%); UTI: 38 (17.6%); Uterine fibroids: 36 (16.7%); Obesity: 31 (14.4%); Hypothyroidism: 27 (12.5%); Arrhythmia: 10 (4.6%); Endometriosis: 8 (3.7%); Type 2 diabetes: 5 (2.3%); Hypertension: 2 (0.9%); Nodular goiter: 2 (0.9%)

Legend: Data are presented as mean ± SD or n (%). AMH = anti-Müllerian hormone; PCOS = polycystic ovary syndrome; PID = pelvic inflammatory disease; UTI = urinary tract infection. Several patients had multiple comorbidities, therefore totals exceed 100%.

**Table 3:** Pregnancy outcomes after palmer-type neosalpingostomy (n = 160).

Outcome	n (%)
**Clinical pregnancy rate (CPR)**	54 (33.8%)
**Intrauterine pregnancy rate (IUPR)**	41 (25.6%) *
**Ectopic pregnancy**	7 (4.4%)
**Miscarriage**	6 (3.8%)
**Intrauterine/live birth pregnancy (ongoing/live birth)**	41 (25.6%)

* IUPR calculated as clinical pregnancies minus ectopic pregnancies and miscarriages.

**Table 4:** Pregnancy outcomes by infertility type and hydrosalpinx laterality (n = 160).

Group	Clinical Pregnancy n (%)	Intrauterine/Live Birth Pregnancy * n (%)	No Pregnancy n (%)	Total
**Primary infertility (n = 84)**	28 (33.3%)	21 (25.0%)	56 (66.7%)	84
**Secondary infertility (n = 76)**	26 (34.2%)	20 (26.3%)	50 (65.8%)	76
**Bilateral hydrosalpinx (n = 85)**	31 (36.5%)	21 (24.7%)	54 (63.5%)	85
**Unilateral hydrosalpinx (n = 75)**	23 (30.7%)	20 (26.7%)	52 (69.3%)	75

* Intrauterine/live birth pregnancy = intrauterine pregnancies, excluding ectopic and miscarriage. No significant differences were observed between groups (Chi-square and Fisher’s exact test, all *p* > 0.05). Odds ratio for clinical pregnancy (unilateral vs. bilateral hydrosalpinx) = 0.77 (95% CI, 0.40–1.49; *p* > 0.05).

**Table 5:** Complications and unfavorable outcomes after neosalpingostomy (n = 160).

Complication	n (%)
**Recurrence of hydrosalpinx**	34 (21.2%)
**Ectopic pregnancy**	7 (4.4%)
**Miscarriage**	6 (3.8%)

**Table 6:** Univariate logistic regression for predictors of intrauterine/live birth pregnancy (n = 160).

Predictor	OR	95% CI	*p*-Value
**Age**	1.01	0.93–1.09	0.823
**AMH**	0.96	0.67–1.38	0.814
**Laterality (bilateral vs. unilateral)**	0.90	0.44–1.84	0.777
**Recurrence**	≈0.00	–	0.999
**Past medical history**	–	–	–
**Adhesions**	0.28	0.13–0.59	**0.001**

**Table 7:** Multivariate logistic regression for predictors of intrauterine/live birth pregnancy (n = 160).

Predictor	OR	95% CI	*p*-Value
**Age**	1.01	0.91–1.13	0.797
**AMH**	0.98	0.61–1.57	0.925
**Adhesions**	0.28	0.13–0.59	**0.001**

**Table 8:** Intrauterine/live birth pregnancy outcomes according to hydrosalpinx severity (n = 160).

Hydrosalpinx Severity	Intrauterine/Live Birth Pregnancy: Yes (n)	Intrauterine/Live Birth Pregnancy: No (n)	Total (n)	Success Rate (%)
**Minor**	33	12	45	73.3
**Moderate**	7	20	27	25.9
**Severe**	9	79	88	10.2

No pregnancy was observed in patients with recurrent hydrosalpinx, whereas the intrauterine/live birth pregnancy rate among those without recurrence was 38.9%.

**Table 9:** Time to clinical pregnancy after Palmer-type neosalpingostomy (n = 160).

**Time After Surgery (Months)**	**Cumulative Incidence of Pregnancy (%)**
**3**	0.6%
**6**	4.4%
**9**	16.2%
**12**	21.9%
**15**	25.6%

Legend: Median time to clinical pregnancy = 9.0 months (IQR 8.0–12.0). Non-pregnant cases were censored at last follow-up.

## Data Availability

The data presented in this study are available on request from the corresponding author. The data are not publicly available due to patient confidentiality.
